# Artificial intelligence derived grading of mustard gas induced corneal injury and opacity

**DOI:** 10.1038/s41598-025-08042-x

**Published:** 2025-07-01

**Authors:** Rajnish Kumar, Devansh M. Sinha, Nishant R. Sinha, Ratnakar Tripathi, Nathan Hesemann, Suneel Gupta, Anil Tiwari, Rajiv R. Mohan

**Affiliations:** 1 Harry S. Truman Memorial Veterans’ Hospital, Columbia, MO USA; 2 Department of Veterinary Medicine & Surgery, College of Veterinary Medicine, University of Missouri, Columbia, MO USA; 3 Mason Eye Institute, School of Medicine, University of Missouri, 1600 E. Rollins St, Columbia, MO 65211 USA

**Keywords:** Artificial intelligence, Cornea, Fibrosis, Sulfur mustard, Pathology, Computational biology and bioinformatics, Corneal diseases

## Abstract

Artificial intelligence (AI) has emerged as a transformative tool in ophthalmology for disease diagnosis and prognosis. However, use of AI for assessing corneal damage due to chemical injury in live rabbits remains lacking. This study aimed to develop an AI-derived clinical classification model for an objective grading of corneal injury and opacity levels in live rabbits following ocular exposure of sulfur mustard (SM). An automated method to grade corneal injury minimizes diagnostic errors and enhances translational application of preclinical research in better human eyecare. SM induced corneal injury and opacity from 401 in-house rabbit corneal images captured with a clinical stereomicroscope were used. Three independent subject matter specialists classified corneal images into four health grades: healthy, mild, moderate, and severe. Mask-RCNN was employed for precise corneal segmentation and extraction, followed by classification using baseline convolutional neural network and transfer learning algorithms, including VGG16, ResNet101, DenseNet121, InceptionV3, and ResNet50. The ResNet50-based model demonstrated the best performance, achieving 87% training accuracy, and 85% and 83% prediction accuracies on two independent test sets. This deep learning framework, combining Mask-RCNN with ResNet50 allows reliable and uniform grading of SM-induced corneal injury and opacity levels in affected eyes.

**Supplementary Information:**

The online version contains supplementary material available at 10.1038/s41598-025-08042-x.

## Introduction

Recent advancements in deep learning algorithms, especially convolutional neural networks (CNNs), have significantly advanced ophthalmology care, particularly in the diagnosis and grading of various ocular diseases. Cutting-edge applications of CNNs in ophthalmology span diverse areas: SCINet, a segmentation and classification interaction network, has demonstrated efficacy in grading arteriosclerotic retinopathy with enhanced accuracy and robustness^1^. Similarly, CNN-long short-term memory (LSTM) models incorporating longitudinal visual field data have shown promise in predicting primary open-angle glaucoma progression^2^. The use of CNNs with longitudinal macular optical coherence tomography angiography (OCTA) imaging has provided a novel approach to detecting glaucoma progression, further emphases the importance of temporal data integration^3^. High diagnostic accuracy has also been achieved in identifying glaucoma in highly myopic populations through tailored CNN architectures^4^. Hybrid models combining CNNs, and recurrent neural networks (RNNs) have advanced diabetic macular edema screening, leveraging complementary strengths of feature extraction and sequential data analysis^5^. ResNet50, VGG19, and InceptionV3 CNN architectures have been applied for retinal vein occlusion diagnosis using fundus fluorescein angiography^6^. Additionally, CNN-based approaches have enabled precise fungal keratitis diagnosis via in vivo confocal microscopy for rapid and accurate infectious disease detection^7^. Machine learning models integrating CNNs have also been employed to establish links between retinal biomarkers and ischemic stroke subtypes and thereby providing insights into systemic disease pathophysiology^8^. Comprehensive frameworks using CNNs have facilitated multi-disease detection from fundus images^9^, while deep learning methods have successfully identified hard exudates and retinal inner layer disorganization in diabetic macular edema for more targeted clinical interventions^10^.

The application of deep learning algorithms for the classification and prediction of corneal pathologies remains underexplored. Sulfur mustard gas (SM) induced corneal injury and opacity presents unique diagnostic challenges and requires reliable grading systems for clinical assessment^11–13^. In this study, we report development of an CNN-based classification system for grading SM-induced corneal injury and opacity in live rabbits using corneal images taken from stereomicroscope. Further, we evaluated the diagnostic performance of the developed model on independent sets of SM-exposed corneas. We investigated the transfer learning method with the highest performance in the classification of SM-injured corneal pathologies, with a focus on diagnosing and detecting the corneal pathology grades. Furthermore, we examined whether it is possible to achieve reliable diagnostic outcomes via a skewed/severely injured corneal image dataset. This work addresses the unmet need for an objective, image-based classification system, gauging SM-induced corneal damage. An overarching goal of the present study is to develop a clinically translatable AI model that supports development of diagnostic tools and medical countermeasures through standardized grading of ocular chemical injury.

## Methods

### SM vapor exposure and corneal imaging

All SM vapor exposures were performed at MRI Global (Kansas City, MO, United States) as previously described^14,15^. Rabbits were anesthetized via intramuscular administration of ketamine (up to 60 mg/kg) and xylazine (up to 5 mg/kg) and given buprenorphine HCl (0.05–0.1 mg/kg) for pain management. After anesthesia, custom goggle was secured around the animals’ head, and eye was exposed to SM vapor inside a chemical hood at a target concentration of 200 mg-min/m³ for 8 min. Following exposure, the goggles were removed after a 2-minute washout period, and both eyes were rinsed with balanced salt solution (BSS) for decontamination. Animals were recovered from anesthesia inside the chemical hood and subsequently transported to a separate facility for long-term monitoring. All animals were monitored for up to 12 months post-exposure to assess the progression of corneal injury. Corneal imaging was performed at day 7, day 14, day 21, and subsequently once every month from 1 to 12 months post-exposure to monitor injury progression.

Rabbit corneal images were captured over this period using a stereomicroscope (Leica MZ16F, Leica Microsystems Inc., Buffalo Grove, IL) equipped with a SpotCam RT KE digital camera system (Diagnostic Instruments Inc., Sterling Heights, MI) to document corneal and ocular damage. Additionally, corneal examinations were performed using a single portable slit-lamp microscope (Kowa SL-15, Torrance, CA) for ocular health and corneal haze assessment^16^.

Imaging was performed by trained research personnel under standardized protocols, with consistent machine settings across all imaging sessions to minimize inter-observer variability. A total of 401 corneal images were collected, including 94 healthy/naïve images, which were subsequently utilized for masking and classification using deep learning algorithms. The animal ethics committee of the University of Missouri, Veterinary Medicine, approved the study, which was conducted in accordance with ARRIVE and ARVO guidelines for the use of animals in research.

### Subjective grading of corneal images

Three ophthalmology researchers graded the images in a blinded manner. This subjective grading/classification was performed based on corneal clinical features listed in Table 1. Representative corneal images corresponding to each severity grade are provided in Supplementary information S1.

**Table 1:** Subjective classification of SM-induced corneal injury based on clinical features.

Clinical feature	Severity of the disease			
	Healthy	Mild	Moderate	Severe
Iris visible	✔	✔	Partial	✖
Corneal haze	✖	✔	✔	✔
Blood vessels- 1–2 mm not reaching the central cornea	✖	✖	✔	✔
Blood vessels- more than 4 mm reaching the central cornea	✖	✖	✖	✔
Epithelial defect	✖	✖	✔	✔

The grading was performed based on the iris visible/not visible, corneal haze, extent of blood vessel invasion (categorized by proximity to the central cornea), and epithelial defects were noted as either absent (✖), partial or present (✔).

### Image preprocessing and augmentation

The training process starts with preprocessing the image data, which involves resizing and augmenting the images. The training images were utilized to develop various CNN models via nested k-fold cross-validation (k = 3, 5, 7, 10) with class-stratified folds. In this approach, the training set was divided into k subsets, with the model being trained iteratively using all but one subset, which was held out for validation to assess the model’s performance^17^. A single imaging setup with consistent magnification setting was used. A minor resolution variability occurred due to unavoidable clinical artifacts such as tear film irregularities, motion blur, and focal adjustments during live animal imaging. All captured images were subsequently resized to 240 × 240 pixels to ensure standardized input dimensions for model training. Image augmentation was then applied, including random zooms of up to 10% and both vertical and horizontal flips, to effectively train and validate the CNN models^18,19^.

### Corneal extraction

The mask region-based CNN (Mask R-CNN) algorithm^20^ was used to train graded corneal images with their corresponding JavaScript object notation (JSON) files. The masks for each image were first drawn manually and saved as JSON files. These were then converted into a readable (binary) format and combined into one under the name using the online labeling tool makesense.ai^21–23^. This training allows the algorithm to accurately identify and segment the region of interest in each image^24^. Once the Mask-R-CNN algorithm was trained, it was applied to mask each image, isolating the corneal area while excluding surrounding or irrelevant regions (noncorneal regions, e.g., eyelids, eyelashes, and specula). After masking, the images were cropped to remove any unwanted black areas, ensuring that only the relevant portion of the image remained for further analysis.

### Development of prediction models

The CNN algorithms were applied to the preprocessed data to create two categories of models: (a) baseline models, which were constructed from the ground up, and (b) models utilizing transfer learning, where pretrained network weights were used, followed by fine-tuning of parameters to enhance model performance. This study employed pretrained networks, namely, VGG16, ResNet101, DenseNet121, InceptionV3, and ResNet50^25–28^. For each architecture, grid-based hyperparameter tuning was conducted, generating multiple models with variations in the number of convolutional blocks, filters, dropout rates, learning rates, dense layers, and training epochs (Table 2). Adam (with weight decay: 1e-5) and stochastic gradient descent (SDG) were used as optimizers, and ‘Rectified linear unit’ (ReLU) and SoftMax were used as the activation functions for the dense and output layers, respectively^29–31^. Final model training was limited to a maximum of 100 epochs with early stopping based on validation loss monitoring.

**Table 2:** Values for hyperparameters investigated for training CNN models for SM-induced corneal pathology classification.

Parameter	Values
# convolutional blocks	1–5
# filters	32, 64, 128
Kernel size	1*1, 2*2, 3*3, 4*4
# dense layers	1–5
# neurons	10-1000
Learning rate	2 × 10^− 4^, 1 × 10^− 4^, 2 × 10^− 3^, 1 × 10^− 3^, 1 × 10^− 2^, 2 × 10^− 2^
Dropout ration	0.3, 0.5, 0.7
# epochs	50, 100
Optimizer	Adam, Stochastic gradient descent

### Statistical measures

Several performance parameters were evaluated to determine the best-performing model between the baseline and pretrained networks. These parameters included accuracy (Acc), precision (Pr), recall (R), the hamming distance (HD), the F1 score, the area under the receiver operating characteristic curve (ROC-AUC), and the area under the precision‒recall curve (PR-AUC). The accuracy, precision, and recall are calculated based on true positive (TP), true negative (TN), false positive (FP), and false negative (FN) values. TP occurs when the model correctly predicts a positive class, whereas TN occurs when the model correctly predicts a negative class. Incorrect predictions are represented by FPs and FNs. The hamming distance is used to compare two binary strings of equal length through XOR operations. The F1 score represents the harmonic mean of precision and recall. The ROC curve was used to assess a model’s ability to differentiate between classes, and the area under the ROC curve was used to plot the true positive rate (sensitivity) against the false positive rate (1-specificity) at various thresholds. The PR-AUC was calculated as an alternative metric which is particularly useful for imbalanced datasets. The PR curve plots precision against recall and used to evaluate the model’s performance in identifying the positive class. Equations (1–4) detail the calculations for these performance metrics, including sensitivity, specificity, precision, and recall, which are fundamental for understanding the classification model’s effectiveness.

1 $$\:Accuracy\:\left(Acc\right)=\:\frac{TP\:+\:TN}{TP\:+\:TN\:+\:FP\:+\:FN}$$

2 $$\:\text{Precision\:}\left(\text{Pr}\right)=\:\frac{TP}{TP\:+\:FP}$$

3 $$\:Recall\:\left(R\right)=\:\frac{TP}{FP\:+\:FN}$$

4 $$\:F1\:score=2*\:\frac{precision\:*\:recall}{precision\:+\:recall}$$

The classifier was developed using the hyperparameter combination that achieved the best performance across most statistical measures during k-fold cross-validation. The final model was selected by comparing the performance metrics of the top baseline-CNN model with those of pretrained networks (VGG16, ResNet101, DenseNet121, InceptionV3, and ResNet50). To evaluate the model’s effectiveness and robustness, it was tested on two independent test sets. Additionally, bootstrap modeling was conducted on the test set to estimate the 95% confidence intervals, ensuring the reliability of the final model.

### Validation using Scheimpflug imaging

Pentacam HR (Oculus Optikgerate GmbH, Wetzlar, Germany), an advanced diagnostic tool that uses Scheimpflug imaging to capture precise measurements of corneal thickness, curvature, anterior chamber depth, and lens opacity, was used to validate randomly selected corneal pathology grades (predicted by the best-performing developed model)^32^.

**Fig. 1:**
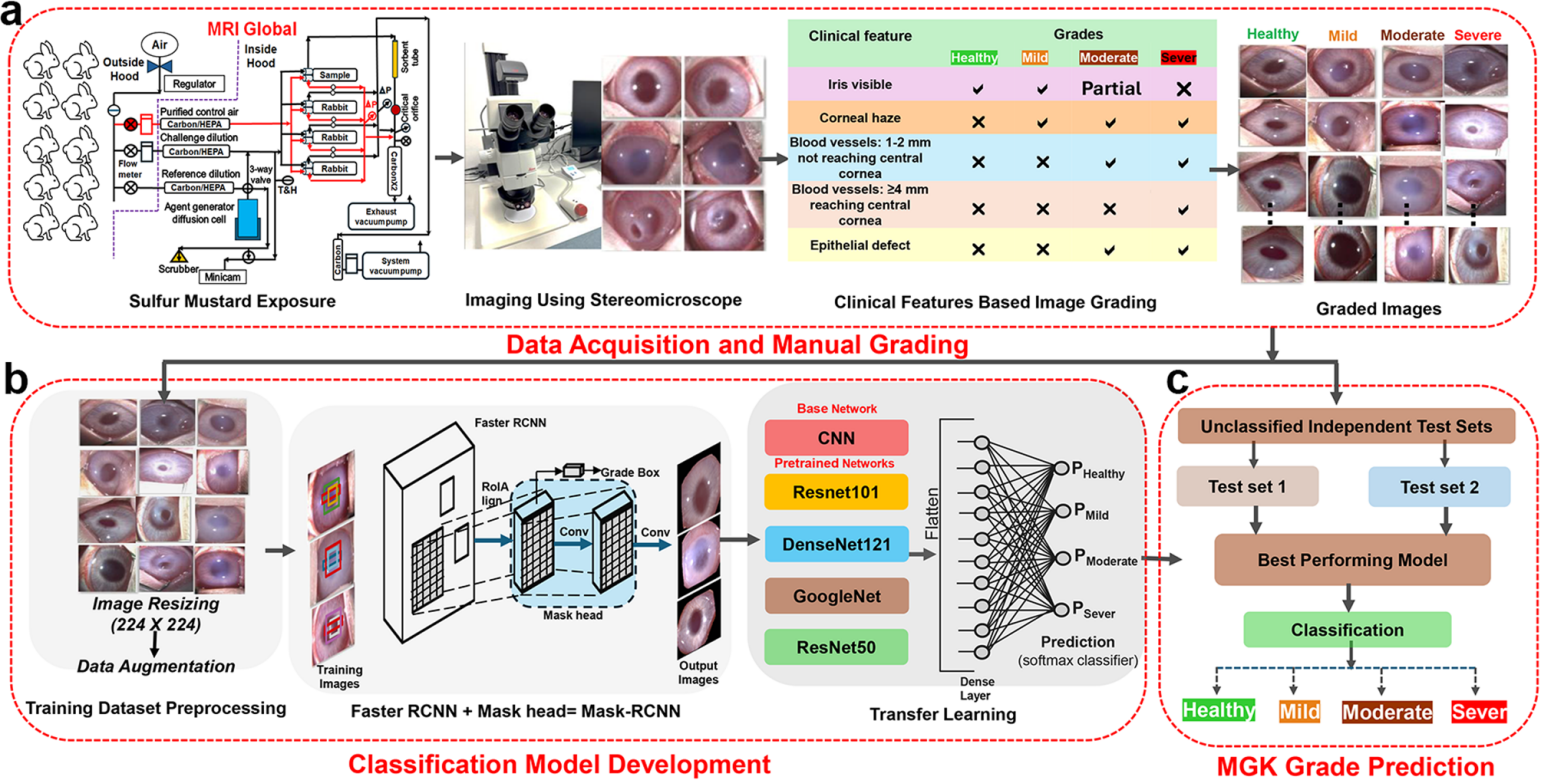
Workflow for the grading of SM-exposed corneas using CNN. The figure illustrates the end-to-end process of developing a deep learning model for grading corneal injury caused by SM exposure. The workflow consists of three main stages: (**a**) data acquisition and manual grading, (**b**) classification model development, and (**c**) SM-induced pathology grade prediction.

The overall methodology, encompassing all key steps from data acquisition to classification, is comprehensively summarized in Fig. 1.

## Results

### Data distribution and augmentation

The image dataset includes four classes, namely, healthy, mild, moderate, and severe corneal pathologies, where each class/grade in the image dataset is represented by 94, 106, 105, and 96 images, respectively. The combined dataset of 401 corneal images was split into training (70%) and two test sets: Test set 1 (T1) and Test set 2 (T2) (15% each). Efforts were made to ensure that both the training and test sets had approximately equal numbers of corneal images across the four classes.

### Optimization of hyperparameters

Various hyperparameter configurations were tested to train multiclass CNN models via 10-fold cross-validation. The hyperparameters used to construct the best-performing CNN models are given in Table 3.

**Table 3:** Architectural configurations and training hyperparameters across various trained models, including a custom baseline-CNN model and pretrained architectures (VGG16, ResNet101, DenseNet121, InceptionV3, and ResNet50).

Parameters	Baseline Model	VGG16	ResNet101	DenseNet121	InceptionV3	ResNet50
Convolutional Blocks	3 (with 2 convolutional layers)	Predefined	Predefined	Predefined	Predefined	Predefined
Filters	First block: 32					
	Second block: 64					
	Third block: 128					
Kernel size	3 × 3					
MaxPooling	2 × 2					
Zero Padding	No	Yes	Yes	Yes	Yes	Yes
Dense Layers	2	2	2	2	2	2
Neurons in Dense Layers	1024,4	1024,4	1024,4	1024,4	1024,4	1024,4
Dropout Ratio	0.5	0.5	0.5	0.5	0.5	0.5
Learning Rate	1.00E-03	1.00E-02	1.00E-04	1.00E-04	2.00E-03	1.00E-04
Epochs	100	100	100	100	100	100
Batch Size	64	64	64	64	64	64
Optimizer	Adam (weight decay: 1e-5)	Adam (weight decay: 1e-5)	Adam (weight decay: 1e-5)	Adam (weight decay: 1e-5)	Adam (weight decay: 1e-5)	Adam (weight decay: 1e-5)

### Classification models for SM-induced corneal pathology

The performance metrics of the models developed using the baseline-CNN, VGG16, ResNet101, DenseNet121, InceptionV3, and ResNet50 methods are given in Table 4.

### Baseline-CNN model

The CNN-based model achieved the lowest performance across both test sets, with overall accuracies of 0.64 on T1 and 0.70 on T2. For the healthy class, the model performed better in Test 2, yielding a sensitivity of 0.87 compared with 0.73 in Test (1) The specificity was also greater for Test (2) However, for the mild class, the sensitivity drastically decreased in Test 2 (0.43), but the specificity was high in both test sets (0.97 and 0.89). Similar trends were observed for the moderate and severe classes. The model’s F1 scores and HD reflected a balance between false positives and false negatives, with HD values of 0.40 indicating modest classification error rates. The micro- and macro-ROC-AUC scores were 0.88 and 0.90, respectively.

### VGG16

VGG16 demonstrated moderate classification performance, with accuracies of 0.75 on T1 and 0.80 on T2. For the healthy class, the sensitivity remained consistent between test sets (0.80 and 0.83), whereas the F1 score and specificity improved slightly in Test 2. The model was strong in classifying the severe class, with F1 scores of 0.87 and 0.71 for T1 and T2, respectively, and a high specificity of 0.96. The ROC-AUC scores were consistently high (0.93 micro- and macrovalues). The PR-AUC scores were slightly better than those of the CNN-based model.

### ResNet101

ResNet101 achieved similar accuracy on both test sets (0.75 and 0.77), with particularly strong performance in the severe class. The F1 score and specificity for severe cases were 0.96 and 0.81 in T1 and T2, respectively. For the healthy class, the sensitivity and specificity were balanced, with values exceeding 0.80 across both test sets. The ROC-AUC and PR-AUC scores indicate comparable performance to that of VGG16, with macro- and micro- ROC-AUC values of 0.92 and 0.93, respectively. The Hamming distance improved over that of the CNN-based model, with an HD value of 0.29.

### DenseNet121

DenseNet121 achieved accuracies of 0.80 on T1 and 0.85 on T2. The sensitivity for the healthy class was particularly high, reaching 0.92 at T2, and the specificity was consistently strong across all classes. Notably, DenseNet121 achieved perfect S-specificity (1.00) for the mild and severe classes in Test 2, along with good F1 scores. HD was relatively low at 0.20. DenseNet121 also achieved the best ROC-AUC scores, with micro- and macrovalues of 0.97 and 0.94, respectively, and high PR-AUC scores (0.84 micro- and 0.89 macrovalues).

### InceptionV3

InceptionV3 had a mixed performance, with accuracy values of 0.77 on T1 and 0.73 on T2. The sensitivity for the healthy class was comparable to that of DenseNet121, but the performance for the mild class was less consistent, with a sensitivity decrease from 0.39 in T1 to 0.40 in T2. The F1 scores for moderate cases were lower compared to other models. HD was slightly higher (0.28), but the ROC-AUC and PR-AUC scores were good but not as high as those of DenseNet121.

### ResNet50

**Fig. 2:**
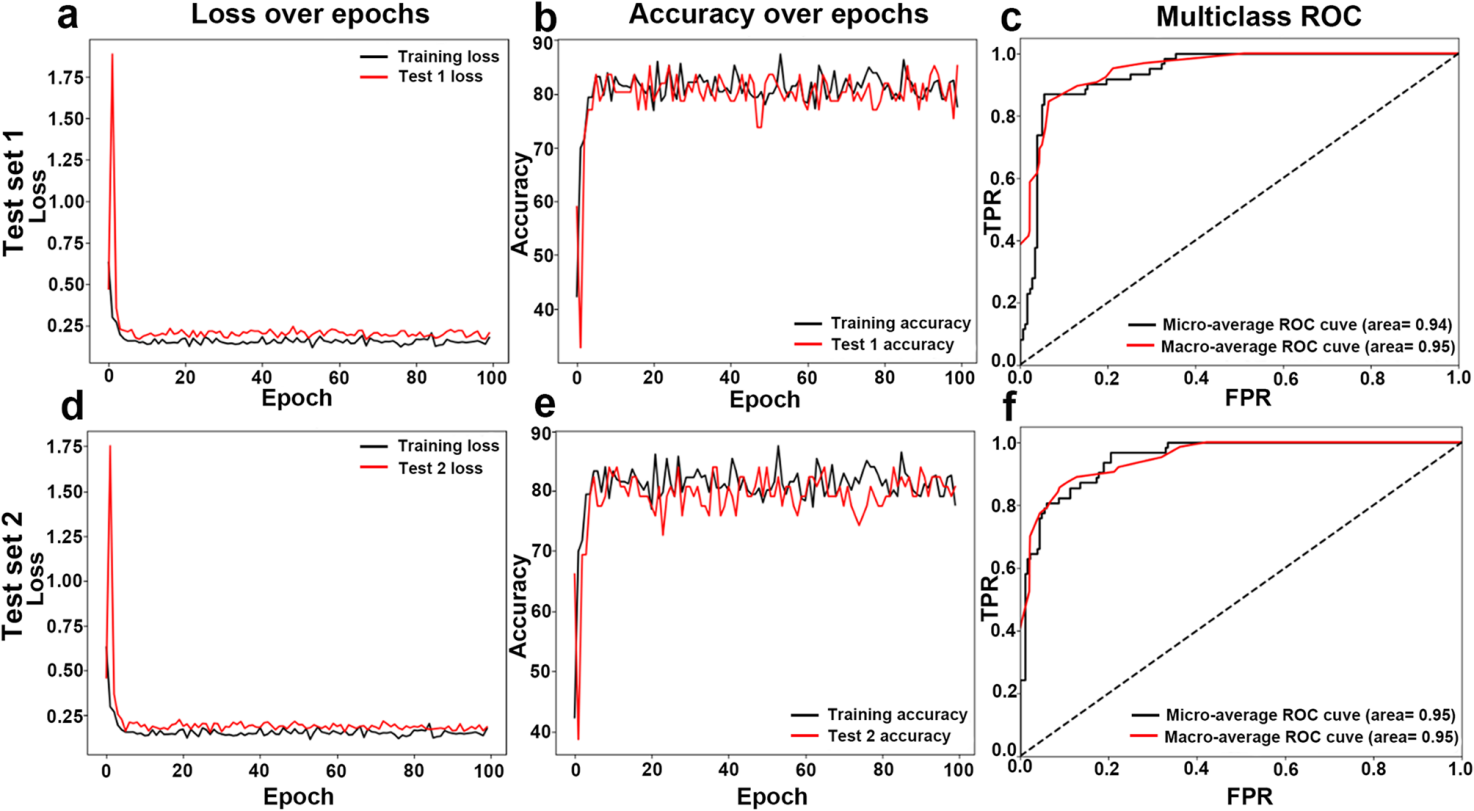
Training and evaluation performance of the ResNet50 model on independent Test Sets 1 (**a**–**c**) and 2 (**d**–**f**). Panels (**a**,** d**) show the loss over epochs, with both training (black) and test set (red) losses decreasing and stabilizing, indicating effective model convergence. Panels (**b**,** e**) display the accuracy over epochs for training (black) and test sets (red), showing high and stable accuracy with minimal overfitting. Panels (**c**,** f**) present multiclass ROC curves for Test Set 1 and Test Set 2, with microaverage and macroaverage ROC-AUC values.

ResNet50 achieved the highest accuracy among all the models, with 0.87 on T1 and 0.85 on T2. This model excelled in classifying healthy and severe cases, achieving perfect sensitivity and specificity for healthy cases in T2 (1.00), and a high F1 score for severe cases (0.94 in T1 and 0.92 in T2). The HD was the lowest among all the models (0.17), which indicates the fewest misclassifications. The model’s ROC-AUC (micro = 0.94; macro = 0.95) and PR-AUC scores (micro = 0.80; macro = 0.84) were also satisfactory (Fig. 2).

**Table 4:** Performance metrics of deep learning algorithms (CNN-Base, VGG16, ResNet101, DenseNet121, inception v3, and ResNet50) across two test sets (T1, T2) for classifying four severity levels of corneal pathology: healthy (H), mild (M), moderate (MO), and severe (S). The performance metrics include the accuracy, sensitivity (Sn), specificity (Sp), F1 score, hamming distance (HD), receiver operating characteristic (ROC)-AUC (area under the curve), and precision recall-area under the curve (PR-AUC). Micro- and Macroaverages for the ROC-AUC and PR-AUC are provided for overall model evaluation.

Model	Performance metrics														
	Accuracy				Sn		Sp		F1		HD (Avg.)	ROC-AUC		PR-AUC	
	Train	T1	T2	Class	T1	T2	T1	T2	T1	T2		Micro	Macro	Micro	Macro
Baseline-CNN	0.64	0.70	0.63	H	0.73	0.87	0.93	0.82	0.76	0.67	0.40	0.88	0.90	0.65	0.72
				M	0.75	0.43	0.97	0.89	0.80	0.52					
				MO	0.50	0.40	0.79	0.84	0.50	0.42					
				S	0.60	0.67	0.81	0.88	0.55	0.63					
VGG16	0.75	0.80	0.74	H	0.80	0.83	0.85	0.91	0.76	0.81	0.29	0.93	0.93	0.82	0.80
				M	0.60	057	0.87	0.90	0.60	0.59					
				MO	0.67	0.50	0.96	0.95	0.74	0.62					
				S	0.91	0.92	0.96	0.84	0.87	0.71					
ResNet 101	0.75	0.77	0.80	H	0.84	0.86	0.93	0.90	0.84	0.77	0.29	0.92	0.93	0.79	0.80
				M	0.45	0.67	0.90	0.88	0.48	0.70					
				MO	0.56	0.69	0.84	0.92	0.57	0.69					
				S	0.77	0.79	0.90	0.96	0.71	0.81					
DenseNet121	0.80	0.81	0.85	H	0.89	0.92	0.95	0.91	0.89	0.84	0.20	0.94	0.97	0.84	0.89
				M	0.55	0.62	0.90	0.95	0.55	0.72					
				MO	0.72	1.00	0.91	0.88	0.74	0.81					
				S	0.92	0.79	0.96	1.00	0.89	0.88					
InceptionV3	0.77	0.73	0.77	H	0.79	0.89	0.88	0.88	0.77	0.83	0.28	0.90	0.91	0.76	0.78
				M	0.39	0.40	0.86	0.94	0.45	0.50					
				MO	0.69	0.67	0.92	0.89	0.69	0.67					
				S	1.00	0.92	0.92	0.92	0.85	0.83					
ResNet50	0.87	0.85	0.83	H	0.90	0.78	1.00	0.98	0.95	0.80	0.17	0.94	0.95	0.80	0.84
				M	0.88	0.79	0.96	0.87	0.88	0.79					
				MO	0.80	0.88	0.91	0.91	0.77	0.83					
				S	0.78	0.92	0.94	0.98	0.74	0.92					

T1 = Test set 1; T2 = Test set 2; H = Healthy; M = Mild; MO = Moderate; S = Severe; Sn = Sensitivity; Sp = Specificity; f1 = Fi score; HD*=Hamming distance (average of T1 and T2); ROC-AUC; Receiver operating characteristic-Area under curve; PR-AUC = Precision recall- Area under curve.

**Fig. 3:**
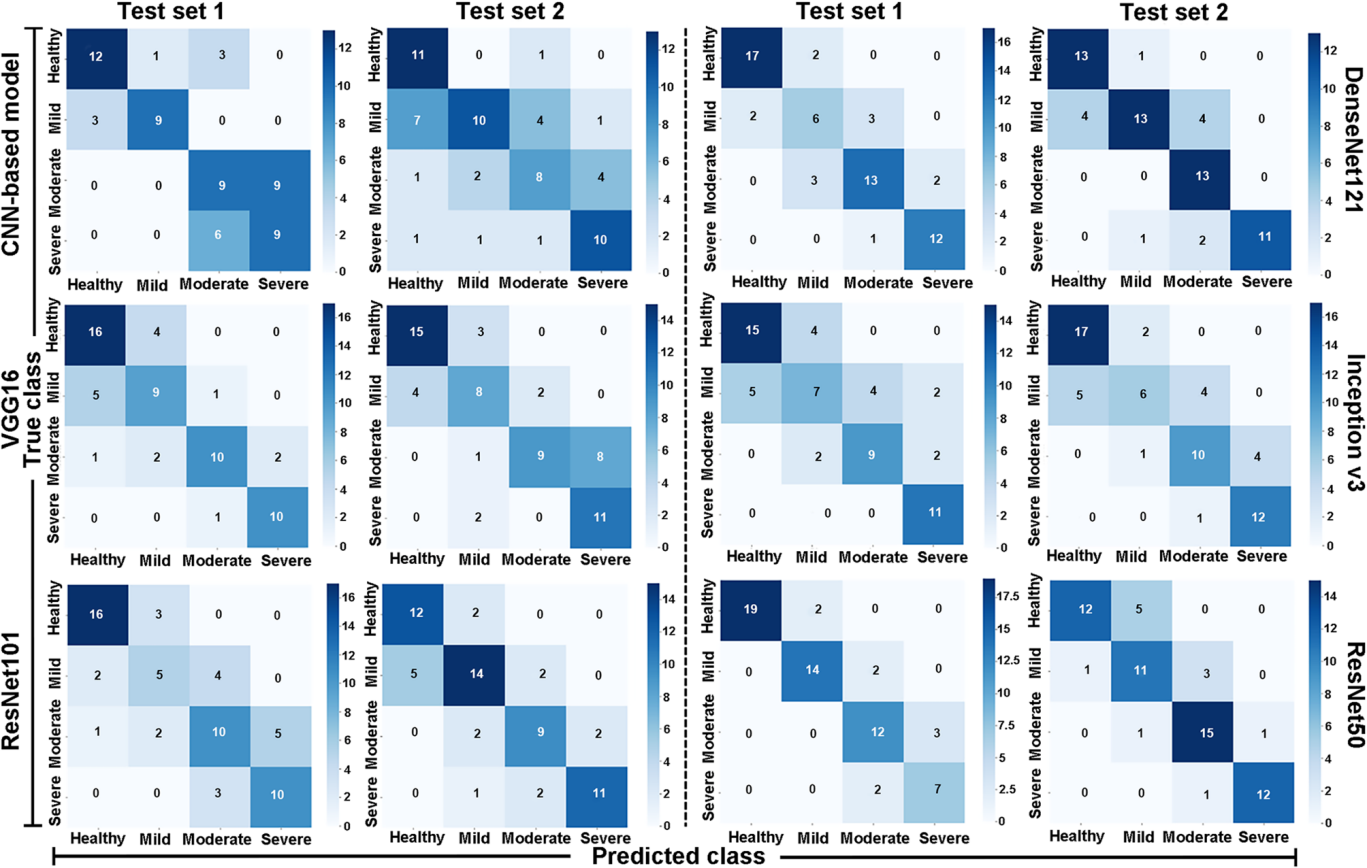
Confusion matrices for six models (Baseline-CNN, VGG16, ResNet101, DenseNet121, InceptionV3, and ResNet50) evaluated on two test sets (Test Set 1 and Test Set 2) for classifying four severity classes: healthy, mild, moderate, and severe. Each cell in the matrix represents the number of true instances (rows) classified as predicted classes (columns). The diagonal values indicate the correct predictions for each class, whereas the off-diagonal values indicate misclassification. The shading represents the relative frequency of correct and incorrect classifications, with darker shades indicating higher counts.

Figure 3 presents confusion matrices for the multiclass classification task on test sets T1 and T2 using Baseline-CNN, VGG16, ResNet101, DenseNet121, InceptionV3, and ResNet50. The Baseline-CNN achieved 39/61 correct classifications in T1 (12 healthy, 9 mild, 9 moderate, and 9 severe) and 39/62 in T2 (11 healthy, 10 mild, 8 moderate, and 10 severe). VGG16 correctly classified 45/61 images in T1 (16 healthy, 9 mild, 10 moderate, and 10 severe) and 43/62 in T2 (15 healthy, 8 mild, 9 moderate, and 11 severe). ResNet101 reached 41/61 in T1 (16 healthy, 5 mild, 10 moderate, and 10 severe) and 46/62 in T2 (12 healthy, 14 mild, 9 moderate, and 11 severe). DenseNet121 accurately classified 48/61 corneas in T1 (17 healthy, 6 mild, 13 moderate, and 12 severe) and 50/62 corneas in T2 (13 each in healthy, mild, moderate, and 11 severe). Inception v3 recorded 42/61 in T1 (17 healthy, 6 mild, 10 moderate, and 9 severe) and 45/62 in T2 (17 healthy, 5 mild, 10 moderate, and 13 severe). ResNet50 demonstrated the highest accuracy, with 52/61 in T1 (19 healthy, 14 mild, 12 moderate, and 7 severe) and 50/62 in T2 (12 healthy, 11 mild, 15 moderate, and 12 severe), outperforming the other models. Overall, the performance of ResNet50 was superior to that of the other algorithms used in this study.

**Fig. 4:**
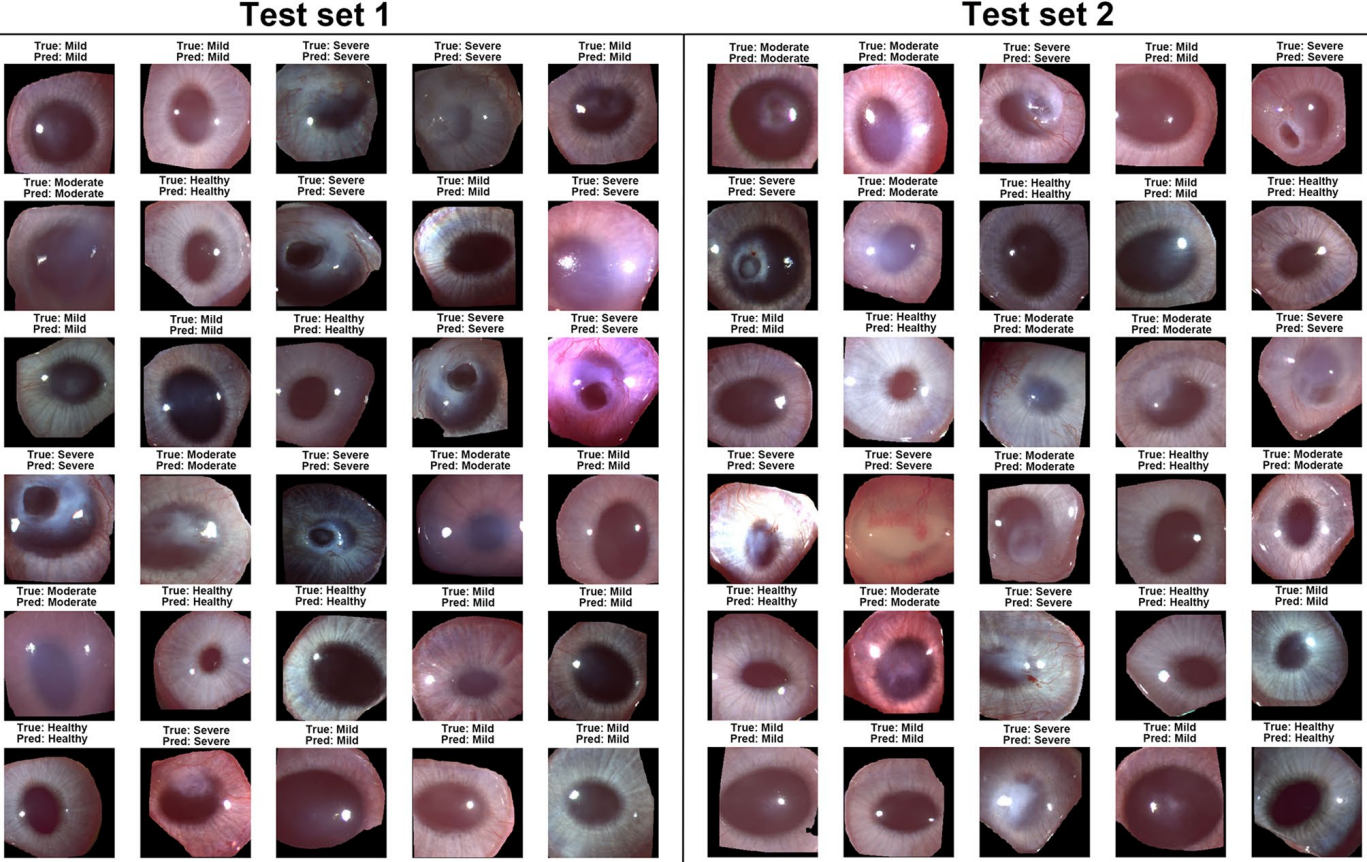
Representative examples of correctly classified corneal pathology grades by ResNet50 across independent test sets. The images in Test Set 1 and Test Set 2 demonstrate the model’s ability to accurately predict corneal health status across various pathology grades, including Healthy, Mild, Moderate, and Severe. For each example, the true label (True) and predicted label (Pred) are shown which illustrates robustness and generalizability of developed model across different datasets. ‘True’ refers to the expert-assigned grade based on manual annotation, while ‘Pred’ indicates the severity grade predicted by the AI model.

**Fig. 5:**
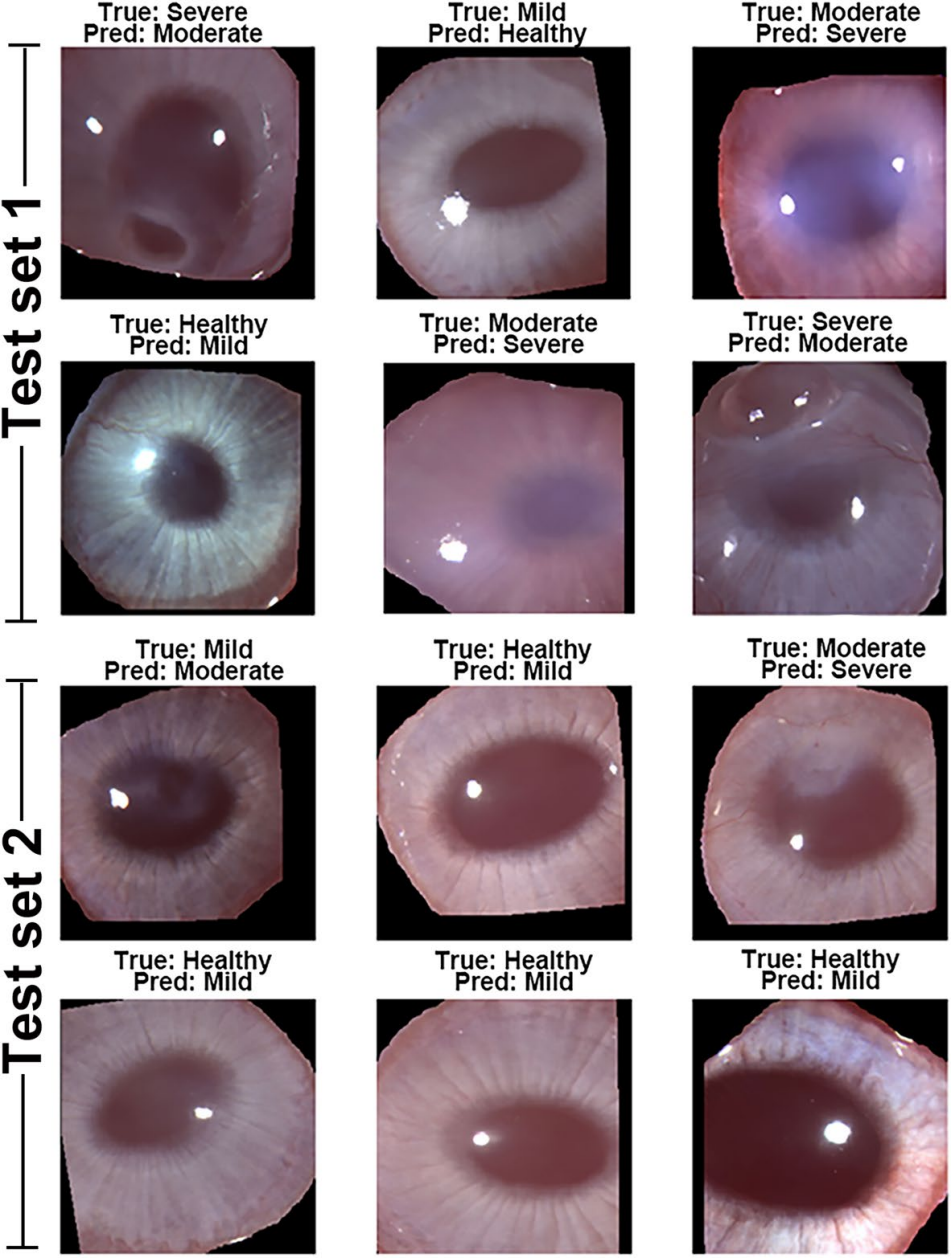
Examples of misclassified corneal pathology grades by ResNet50 across independent test sets. The figure illustrates cases where the model predictions (Pred) did not align with the true labels (True) for different corneal pathology grades in Test Set 1 and Test Set 2.

**Fig. 6:**
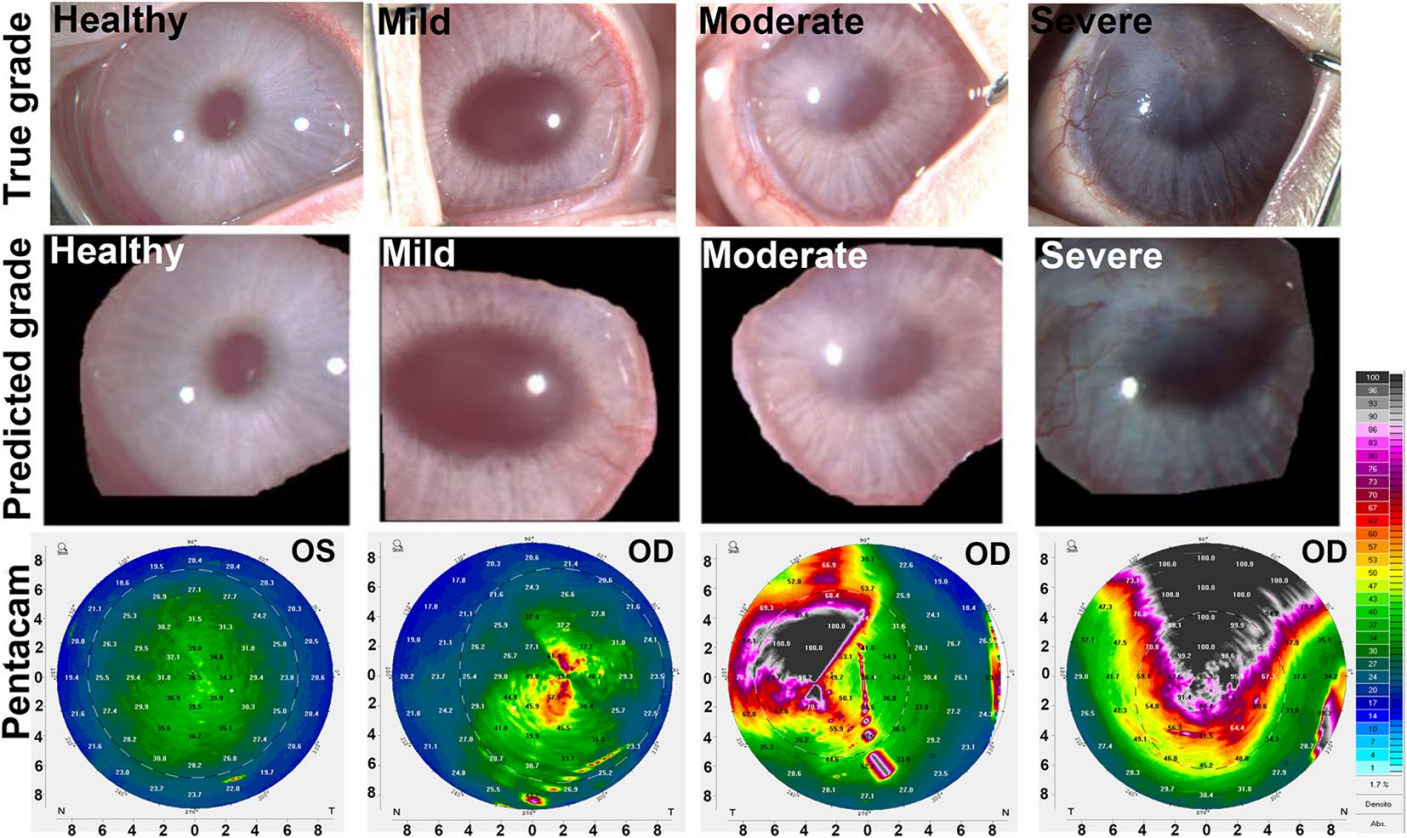
Demonstration of true and predicted corneal pathology grades for SM-exposed rabbit corneas. The first row shows the true pathology grades (healthy, mild, moderate, and severe), whereas the second row displays the grades predicted by the best-performing ResNet50 model. The third row presents corneal density heatmaps generated via Pentacam HR, validating the model’s predictions by illustrating corresponding changes in corneal density for each grade.

The representative examples of accurately classified corneal pathology grades by ResNet50 across independent test sets are shown in Fig. 4. Whereas Fig. 5. Depicts the examples of misclassified corneal grades. Furthermore, randomly selected images (one from each grade) with classes predicted by ResNet50 were used to further validate the results via Scheimpflug imaging (using Pentacam HR), as shown in Fig. 6.

## Discussion

This study demonstrates the effectiveness of AI models, particularly CNN-based architectures, in classifying corneal pathology grades following SM exposure. ResNet50 demonstrated robust performance in classifying corneal pathology grades across independent test datasets. The model was validated using two test sets, encompassing a diverse range of corneal health statuses: Healthy, Mild, Moderate, and Severe. Examples of correctly classified cases are shown in Fig. 4, with true labels and model predictions provided for comparison. The results show ResNet50’s superior performance among the models tested, achieving the highest accuracy across two independent test sets, with 52/61 correct classifications in T1 and 50/62 in T2 (Fig. 3). The misclassified corneal pathology grades are shown in Fig. 5. The misclassification was predominantly limited to adjacent pathology grades. This may indicate that the model encountered some challenges in distinguishing subtle morphological differences between neighboring grades, likely due to overlaps in feature space or variations in image quality, such as lighting, glare, or corneal texture. Additionally, a few training images exhibited some epithelial defects, which may likely have contributed to occasional underestimation of severity. Expanding the dataset to include a more balanced representation of injury-type may enhance model performance in future studies. Nevertheless, the overall performance of ResNet50 in identifying four corneal pathological grades (healthy, mild, moderate, and severe) with high accuracy demonstrates its ability to differentiate severity levels in eye. This will have a valuable clinical application in establishing SM-related corneal pathology.

In addition to ResNet50, DenseNet121 and InceptionV3 also displayed good performance, achieving accuracies of 48/61 and 42/61 in T1 and 50/62 and 45/62 in T2, respectively (Fig. 3). Although VGG16 and ResNet101 achieved moderate accuracy levels (75–80%), the baseline-CNN model performed lower overall, which was the deciding factor in this study to explore deeper and more sophisticated pretrained transfer learning CNN architectures for nuanced classification tasks. These findings provide a proof-of-concept that CNN-based architectures, particularly ResNet50, can be developed as supportive tools for standardized grading of corneal pathology levels in humans following SM injury, pending further validation in human clinical datasets.

The study’s approach is notable for applying transfer learning algorithms to analyze complex stereomicroscope images in an SM-induced corneal injury model. By utilizing clinically relevant parameters in a CNN-based pipeline for grading SM-exposed corneal images, this study not only enhances experimental design and outcome prediction in animal studies but also translates findings into a framework that aligns with human clinical standards. This approach leverages Mask-RCNN and CNN-based models, incorporating both baseline and transfer learning architectures to classify and grade the severity of corneal pathology. Importantly, the methodology encompasses analysis of the entire corneal image, including peripheral regions and the limbus, which enables detection of a broader range of corneal pathologies and enhances diagnostic comprehensiveness.

Compared with other models, performance of ResNet50 was distinguished by consistently high metrics across multiple evaluation measures in this study. With accuracy scores of 0.87 on T1, 0.85 on T2, and 0.83 on the training set, ResNet50 showed exceptional ability to distinguish all four grades, with high specificity in both independent test sets (T1 = 91–100%; T2 = 87–98%) (Table 4). Additionally, ResNet50’s loss plot over the epochs, accuracy plots, and ROC-AUC (T1 = micro- ROC-AUC of 0.94, macro- ROC-AUC of 0.95; T2 = micro-ROC-AUC of 0.95, macro- ROC-AUC of 0.95) scores (Fig. 2) demonstrate strong class discrimination, which is essential for severity classification tasks in clinical practice.

DenseNet121, while also demonstrated competitive performance, exhibited slightly higher HD (0.20) compared to ResNet50, which indicates more misclassifications. The HD of ResNet50 (0.17), which is the lowest among all the models, reflects fewer classification errors (Table 4). The robustness and adaptability of ResNet50 in maintaining high accuracy across various severity classes position it as a promising tool for practical deployment for conditions related to corneal injuries.

The validation of ResNet50’s predictions using Scheimpflug imaging with Pentacam HR further supports the model’s clinical applicability (Fig. 6). The corneal density maps generated by Pentacam HR, reflecting structural changes and opacity, qualitatively aligned with the AI-predicted severity grades which support utility of a developed model in preclinical research. Corneal images collected from Pentacam HR were not utilized for model training but used to assess whether AI-based clinical predictions match with clinical conditions detected by the Pentacam HR technology which provides precise measurements of location and degree of corneal opacity in eyes of human patients in a color-coded manner.

The optimized AI-based model shows promise, its performance in predicting corneal opacity and damage from toxic agents beyond sulfur mustard is required to be validated in broader contexts. Assessing the performance of developed model across a broad spectrum of chemically-induced corneal injuries in patient’s demographics require additional and diverse clinical imaging dataset. Future studies involving larger and more heterogeneous corneal image dataset is essential to fully evaluate the model’s generalizability and robustness towards all chemically injured eyes. Nevertheless, this study addresses a clinical challenge through the corneal image augmentation, rigorous cross-validation, and transfer learning. Moreover, the manual grading process, conducted by three independent clinicians to establish baseline reliability, introduces a degree of subjectivity, which may lead to variability in the ground truth labels. Furthermore, the segmentation process for Mask-RCNN training is labor intensive and susceptible to human error, especially given the potential for annotator fatigue when large image datasets are labeled.

The developed AI-based model achieved an accuracy of approximately 87%, which is comparable to performance benchmarks reported for other ocular diagnostic models^33–40^. This performance of ResNet50 architecture reflects the inherent complexity of classifying subtle variations in corneal pathology. This study also provides a systematic evaluation of CNN-based approaches for grading corneal pathology severity, with ResNet50 emerging as the most effective architecture across independent test sets. Incorporating larger and more diverse corneal image datasets in future work may further enhance model performance and generalizability.

This study employed sulfur mustard-induced mustard gas keratopathy (MGK), characterized by pathological features such as ocular chronic inflammation, recurrent corneal epithelial erosions, stromal scarring, haze, corneal edema/swelling, corneal ulcer, and corneal neovascularization, which are also hallmarks of common corneal conditions seen in patients of infectious keratitis, keratoconus, and dry eye diseases. These clinical parallels suggest that AI models trained on MGK features may have broader relevance particularly in preclinical translational research field. Nevertheless, additional studies are warranted to develop general and disease-specific AI-based models for better clinical diagnosis and treatment.

## Conclusions

By applying advanced CNN models to complex ocular images and focusing on corneal pathology in an animal model, this study may contribute to bridging preclinical research and clinical applicability. The ResNet50 model’s robust metrics and adaptability make it a strong candidate for applications requiring precise multiclass classification, and its demonstrated success offers potential pathways to address SM-induced corneal pathology through early and reliable AI-assisted diagnosis.

## Electronic supplementary material

Below is the link to the electronic supplementary material.

Supplementary Material 1

## Data Availability

The data presented in this study are available on https://github.com/dsinha12345/CPC. The Raw data are available from the corresponding author upon request.

## Declarations

### Disclaimer

The contents do not represent the views of the U.S. Department of Veterans Affairs or the United States Government.
