# Supplementary material for: Artificial intelligence derived grading of mustard gas induced corneal injury and opacity
Source: Sci Rep. 2025 Jul 1;15:20359. doi: 10.1038/s41598-025-08042-x (PMC12216909; doi:10.1038/s41598-025-08042-x)
Supplement: Supplementary file 1 — Supplementary Material 1 [file 41598_2025_8042_MOESM1_ESM.docx]

**Supplementary Information**

**Artificial intelligence derived grading of mustard gas induced corneal injury and opacity**

Rajnish Kumar^1,2^, Devansh M. Sinha^2^, Nishant R. Sinha^1,2,3^, Ratnakar Tripathi^1,2^, Nathan Hesemann^1,3^, Suneel Gupta^1,2^, Anil Tiwari^2^, Rajiv R. Mohan^1,2,3*^

^1^Harry S. Truman Memorial Veterans’ Hospital, Columbia, Missouri, USA

^2^Departments of Veterinary Medicine & Surgery, College of Veterinary Medicine, University of Missouri, Columbia, Missouri, USA

^3^Mason Eye Institute, School of Medicine, University of Missouri, Columbia, Missouri, USA

***Corresponding author**

Rajiv R. Mohan, MSc., Ph.D., FARVO, FAAAS

Curators’ Distinguished Professor of Ophthalmology and Molecular Medicine

University of Missouri, 1600 E. Rollins St, Columbia, MO 65211, United States.

E-Mail: MohanR@health.missouri.edu


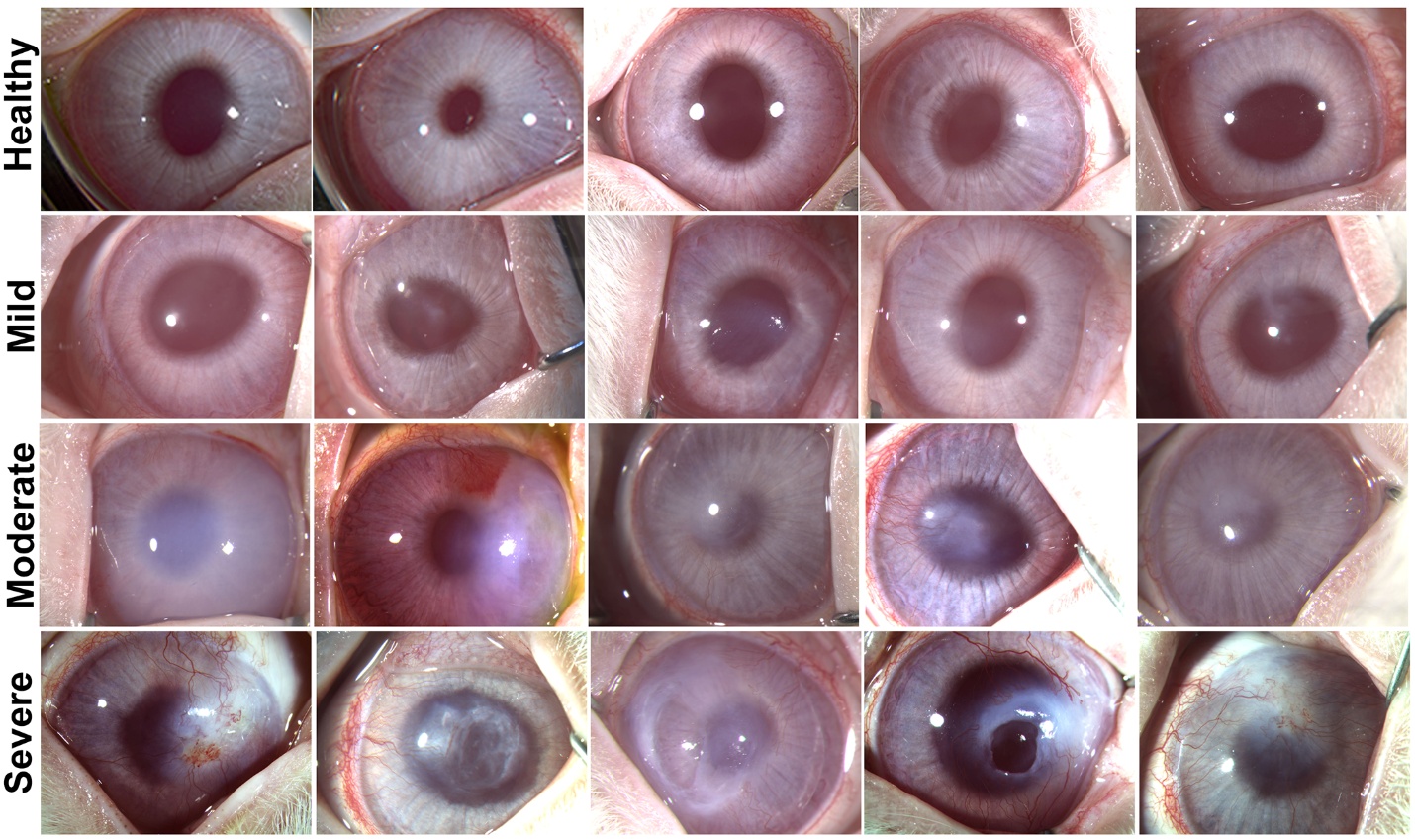


**Fig. S1.** Representative stereomicroscope images illustrating corneal pathology grades post sulfur mustard exposure. Clinical images from live rabbit eyes were captured using stereomicroscope and grouped by severity: Healthy, Mild, Moderate, and Severe. Healthy corneas show clear, transparent stroma with visible iris details. Mild pathology exhibits subtle haze and no or minor vascularization. Moderate cases display increased stromal opacity, vascular encroachment, and visible surface irregularities. Severe pathology shows dense haze, vascular invasion, and obscured iris details. These images provide visual references for the grading scheme used in this study.
